# Sequential exploration in the Iowa gambling task: Validation of a new computational model in a large dataset of young and old healthy participants

**DOI:** 10.1371/journal.pcbi.1006989

**Published:** 2019-06-13

**Authors:** Romain Ligneul

**Affiliations:** Donders Center for Cognitive Neuroimaging, Nijmegen, The Netherlands; Brain and Spine Institute (ICM), FRANCE

## Abstract

The Iowa Gambling Task (IGT) is one of the most common paradigms used to assess decision-making and executive functioning in neurological and psychiatric disorders. Several reinforcement-learning (RL) models were recently proposed to refine the qualitative and quantitative inferences that can be made about these processes based on IGT data. Yet, these models do not account for the complex exploratory patterns which characterize participants’ behavior in the task. Using a dataset of more than 500 subjects, we demonstrate the existence of sequential exploration in the IGT and we describe a new computational architecture disentangling exploitation, random exploration and sequential exploration in this large population of participants. The new Value plus Sequential Exploration (VSE) architecture provided a better fit than previous models. Parameter recovery, model recovery and simulation analyses confirmed the superiority of the VSE scheme. Furthermore, using the VSE model, we confirmed the existence of a significant reduction in directed exploration across lifespan in the IGT, as previously reported with other paradigms. Finally, we provide a user-friendly toolbox enabling researchers to easily and flexibly fit computational models on the IGT data, hence promoting reanalysis of the numerous datasets acquired in various populations of patients and contributing to the development of computational psychiatry.

## Author summary

The ability to perform decisions and learn from their outcomes is a fundamental function of the central nervous system. In order to maintain their homeostasis and maximize their biological fitness, organisms must maximize rewards and minimize punishments. Yet, pure exploitation often leads to suboptimal solutions. In order to discover the best course of action, organisms must also explore their environment, especially when this environment is complex or volatile. Here, we dissected exploratory strategies in one of the most classic decision-making paradigms of cognitive neuroscience. First, we found that humans tend to sample sequentially the space of possible actions. Second, we developed a new mathematical model better able to predict trial-by-trial choices, by articulating this sequential exploration mechanism with random exploration and exploitation. Third, we showed that sequential exploration reduces across lifespan, a result which might be explained by specific neuroanatomical or neurochemical changes associated with normal aging. Together, these findings may contribute to a better understanding of exploratory behaviors and a better assessment of their disruption in a wide range of neuropsychiatric conditions.

## Introduction

**Fig 1:**
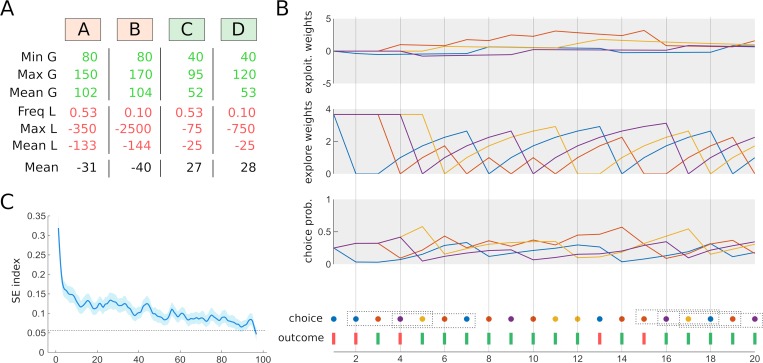
Directed exploration in the IOWA Gambling Task. (A) In the IGT, participants must sample 4 decks of card associated with gains and losses whose magnitudes vary in a probabilistic manner. Unbeknownst to participants, decks C and D are advantageous despite offering smaller gains, because the losses are respectively very low or rarely encountered. The columns below each deck report the empirical minimum, maximum and average magnitude of gains (all decks have a 100% gain probability), the frequency, maximum and mean magnitude of losses, as well as the overall expected value. (B) Here, we propose a new computational model accounting for trial-by-trial choices in the IGT. The “Value and Sequential Exploration” (VSE) model consist of two learning modules tracking respectively the net amount of money generated (exploitation weights, top) and the exploration weights of each deck (dependent upon the time elapsed since the last selection of that deck). These two weights are then summed and transformed into a probability of choosing each deck through a classical softmax. As such, the model implements a straightforward arbitration between reward- and information-seeking drives. (C) The VSE architecture was justified by the discovery of a peculiar choice pattern in the IGT, that it can reproduce for some combination of parameters. Namely, the probability of choosing 4 different decks within 4 consecutive trials (a pattern referred to as the SE index) was largely above chance levels, especially in the beginning of the task.

Many neuropsychiatric disorders are associated with alterations of learning and decision-making. Standardized cognitive paradigms are thus increasingly used to improve diagnosis and evaluate the response to treatments. Developed 25 years ago [1], the Iowa Gambling Task (IGT) remains one of the most popular tools used for this purpose in clinical settings (Fig 1A). Over the years, it has been applied more or less successfully to many populations such as patients suffering from brain lesions, Parkinson disease, behavioral or substance addictions, mood disorders, personality disorders, etc. Although its reinforcement schedule confounding risk and punishment processing can be criticized, the IGT thus remains of considerable importance for the development of scalable methods in cognitive science and in the emerging field of computational psychiatry.

The classical analysis strategy for IGT data results in a crude estimate of decision-making deficits. Based on the relative preferences for “advantageous” decks (typically offering small gains but even smaller losses) over “disadvantageous” decks (typically offering big gains but even bigger losses), this approach does not leverage the full potential of the IGT. From a computational viewpoint, the IGT is indeed a highly complex task engaging value-based learning, risky decision-making, working memory and—as we shall see—different types of exploration. A series of computational models have been developed to better isolate these components, thereby offering clinicians and clinical neuroscientists more precise analytical tools to assess the cognitive profile of their patients. So far, computational neuroscientists interested in the IGT have mainly focused their efforts on the value-based learning and decision-making components of the task. Accordingly, the Expected Value (EV) or the Prospect Valence Learning (PVL, PVL-Delta) algorithms aim at capturing the non-linear and/or asymmetric decision weights associated with gains and losses [2,3]. More recently, the Value Plus Perseveration (VPP) model was developed to capture systematic perseveration or alternation tendencies across successive decisions [4]. However, the VPP model can be criticized for its high number of free parameters (8) relative to the number of trials (100) as well as for the uncertain cognitive validity of its perseveration module [5]. Finally, the last model proposed to date—termed Outcome Representation Learning (ORL)—also encompasses a perseveration module but tracks and weights separately (at the time of decision) the magnitude and the probability of positive and negative outcomes [6]. While the ORL was shown to perform better than alternative models, this latter feature is rather unlikely from the perspective of behavioral economics, as it implies that the decision-maker never combines reward magnitude and reward probability into an estimate of reward expectancy.

Here, we adopted another modeling strategy leveraging the existence of directed exploration (DE) in the class of multi-armed bandit tasks to which the IGT belongs [7–9]. Wilson and colleagues defined directed exploration as “a strategy in which choices are explicitly biased toward information”, as opposed to undirected (or random) exploration corresponding to a “strategy in which decision noise leads to exploration by chance” [10]. Thus, DE constitutes an “umbrella term” as it can refer to any regular choice pattern which: (i) maximize information about available options, (ii) cannot be readily explained by participants’ sensitivity to gains and losses. In the context of the IGT, a straightforward DE strategy is to allocate an “exploration bonus” to the behavioral options which have been sampled less often or less recently than others. This mechanism entails the sequential selection of all available option irrespective of their value or uncertainty, hence resulting into a specific choice pattern: namely, the tendency to select the four available decks over 4 consecutive trials (hereafter referred to as the Sequential Exploration (SE) index). Exploration bonuses and sequential exploration have a long history in the reinforcement learning literature, as they were already proposed in the seminal book of Sutton and Barto (Dyna-Q+ algorithm) [11]. They are also conceptually related to the optimistic initialization techniques used to ensure that all available options available to a decision-maker are sampled before settling on one of them [12].

Thus, we designed a compact computational architecture termed Value and Sequential Exploration in order to simultaneously capture exploitation, random exploration and sequential exploration in the IGT using 5 parameters. The core innovation of this new model is to articulate two types of choice strategies: a reward-seeking strategy shaped by reinforcement history and an information-seeking strategy shaped by choice history (Fig 1B). Governed by a value sensitivity and a decay parameter, the former module reacts to the gains and losses delivered during the task. As such, it resembles to the PVL model except that it includes no loss aversion parameter. The latter module relies on an exploration bonus specific to each participant—which can be either positive or negative depending on whether a given participant tends to explore or avoid options which have not been sampled recently—as well as on a learning rate—which determines how fast the exploration weight of a given deck goes back to the initial value of the bonus after having been sampled. Unlike other forms of directed exploration (such as uncertainty-dependent exploration), the exploration weights of the VSE model are totally independent of gains and losses. Different parameter combinations are thus able to reproduce the full range of possible SE indexes, from 0 to 100% frequency (see Methods for more details).

In order to demonstrate the superiority of the VSE model over the alternatives mentioned above (EV, PVL, PVL-delta, VPP, ORL), we reanalyzed a multi-study dataset of 504 participants who passed the 100 trials version of the IGT [13] (Fig 1A). State-of-the-art model comparison, simulation, as well as model and parameter recovery analyses were performed [14]. Second, in order to evaluate the cognitive validity to our model and illustrate its heuristic value, we focused on the data corresponding to the study of Wood and colleagues testing IQ-matched groups of old and young adults [15]. Indeed, it was recently shown that directed exploration diminishes across lifespan [16,17], so that the exploration bonus of older participants should be smaller than that of young participants. Third, we provide an open-source, user-friendly Matlab toolbox which has been developed to obtain the current results and which shall enable researchers who are not experts in computational models to re-analyze IGT data using both our new model and previous ones (https://github.com/romainligneul/igt-toolbox).

## Results

### Presence of sequential exploration in the IGT

First, we evaluated whether sequential exploration (SE) occurred in the Iowa Gambling Task. To this end, we computed the “SE index” probing situations in which participants selected each of the four different decks over four successive trials using 25 independent consecutive quadruplets: e.g. 1–4, 5–8, etc. In the 504 subjects dataset, we observed such pattern 1400 times (11.1%) while only 1182 occurrences would be expected under random exploration (i.e. 9.38%, binomial test: p<10^−10^). Note that this test is highly conservative, as reward-maximization strategies bias choices towards the most valuable decks. Accordingly, a permutation approach in which trials were shuffled in time for each subject independently (total number of permutations: 5000) showed that the actual chance level was at 6.0%.

The target pattern was much more frequent in the first 20–30 trials of the task and it continuously declined as subjects formed more precise representation of each desk value and learned to exploit the reward structure of the task (Fig 1C). Interestingly, SE had a complex but strong relationship with decision-making performances in the IGT. A general linear model (GLM) analysis indicated that subjects with the highest overall performance had lesser SE indexes (linear effect: t(1,501) = -3.40, p<0.001), presumably due to the fact that these subjects needed less exploratory trials to figure out the reinforcement structure of the task. However, we also observed low SE indexes in the worst subjects, presumably due to maladaptive perseveration, which translated into a significant quadratic relationship between SE and performance (t(1,501) = 2.13, p = 0.034). Overall, the analysis of the SE index justified the development of a computational model capturing this important and previously overlooked exploration strategy in the IGT.

### Value plus Sequential Exploration (VSE) model

Like all previous models, the Value and Sequential Exploration (VSE) architecture updates “exploitation weights”, which keep track of the recent trends in gains and losses associated with each deck. However, the VSE model also updates on each trial the “exploration weights” associated with each deck. Depending solely upon the choice history, this exploration module was designed to capture the dynamics of sequential exploration observed in the IGT. On each trial, exploitation and exploration weights are simply summed into a composite value before being transformed by a conventional softmax step into choice probabilities. Hereafter, we describe the equations and the parameters which fully characterize VSE.

The exploitation module is directly inspired by the PVL model (Steingroever et al., 2013) although it includes no “loss aversion” parameter. A value sensitivity parameter controlled by θ (bound between 0 and 1) is instead applied separately to wins and losses.

$$v(t)={Gain(t)}^{\theta }-{Loss(t)}^{\theta }$$ (1)

On each trial, the exploitation weight of each desk d is updated according to the following equations:

$${Exploit}^{d}(t+1)={Exploit}^{d}(t)*\Delta +v(t)$$ (2.A)

$${Exploit}^{d}(t+1)={Exploit}^{d}(t)*\Delta$$ (2.B)

Eq (2.A) controls the update of the deck chosen, by adding the feedback just experienced to the (decayed) value of this deck. Eq (2.B) controls the update of unchosen decks, whose exploitation weight progressively returns to 0 at a rate controlled by the decay parameter Δ (bound between 0 and 1). Note that a decay of 1 indicate that exploitation weights are integrated over all previous trials, while a decay parameter of 0 indicate that subjects’ decisions rely mostly on the most recent outcomes.

The main innovation provided by VSE consists in modeling sequential exploration in the IGT. Exploration weights reflect the attractiveness of each deck as a function of the number of trials for which the deck has not been selected. Exploration weights are agnostic regarding the monetary feedbacks experienced in the task. As such, they capture a pure information-seeking process, hence contrasting with Bayes-based uncertainty-minimization algorithms as well as the exploration modeled by the softmax or e-greedy rules [8]. Exploration weights are controlled by the following equations:

$${Explore}^{d}(t+1)=0$$ (3.A)

$${Explore}^{d}(t+1)={Explore}^{d}(t)+\alpha *(\varphi -{Explore}^{d}(t))$$ (3.B)

Eq (3.A) controls the update of exploration weights for the selected deck, which fall to zero as soon as the outcome of that deck is sampled. Eq (3.B) controls the update of unselected decks, which is governed by a simple delta-rule. The learning rate α (bound between 0 and 1) determines at which speed the exploration weights return to their initial value, defined by a free parameter termed “exploration bonus” or *φ* (unbounded). A positive exploration bonus implies that the agent is attracted by decks which have not been explored recently, whereas a negative exploration bonus implies that the agent tends to favor familiar decks. Therefore, the exploration bonus φ directly reflects the strength of sequential exploration, so that a more positive value will translate into a higher probability of reproducing the aforementioned pattern of 4 different choices over 4 consecutive trials.

$$P(Choice=d)=\frac{e^{{(Explore}^{d}+{Exploit}^{d})*Consistency}}{\sum_{i=1}^{4}e^{{(Explore}^{i}+{Exploit}^{i})*Consistency}}$$

Finally, Eq (4) models decision-making as a stochastic process controlled by the consistency parameter C: a higher C value indicates that choices are strongly driven by the composite values derived from Eqs 1–3, whereas a C value of zero indicate random selection of each deck. Note that C results from the transformation of an inverse temperature β (bound between 0 and 5), in order to match PVL, PVL-Delta, VPP and ORL models (where C = 3^β^ -1 as well).

Crucially, the architecture of VSE can account for purely random exploration (β = 0), for purely value-based exploitation (β>>0, θ>0 and *φ* = 0), for purely directed exploration (β>>0, θ = 0, and *φ*>0) and for a mixture of value-based exploitation, directed exploration and random exploration. Note that under purely directed exploration, the model predicts that the 4 decks should be successively selected in a cyclical manner, during the whole task (e.g. 3,2,4,1,3,2,4,1,3,etc.), hence reflecting exactly the definition of the SE index. Indeed, in such case, the deck with the highest exploration weight is always the deck which has not been selected for the longest period of time. Finally, a variant of the VSE model including a loss aversion parameter was also tested (VSE+LA). In this model, the update of exploitation weights is therefore identical to that of the PVL model, augmented with the sequential exploration module of the VSE (see S2 Fig for model comparison analyses including VSE+LA).

### Model comparison

**Fig 2:**
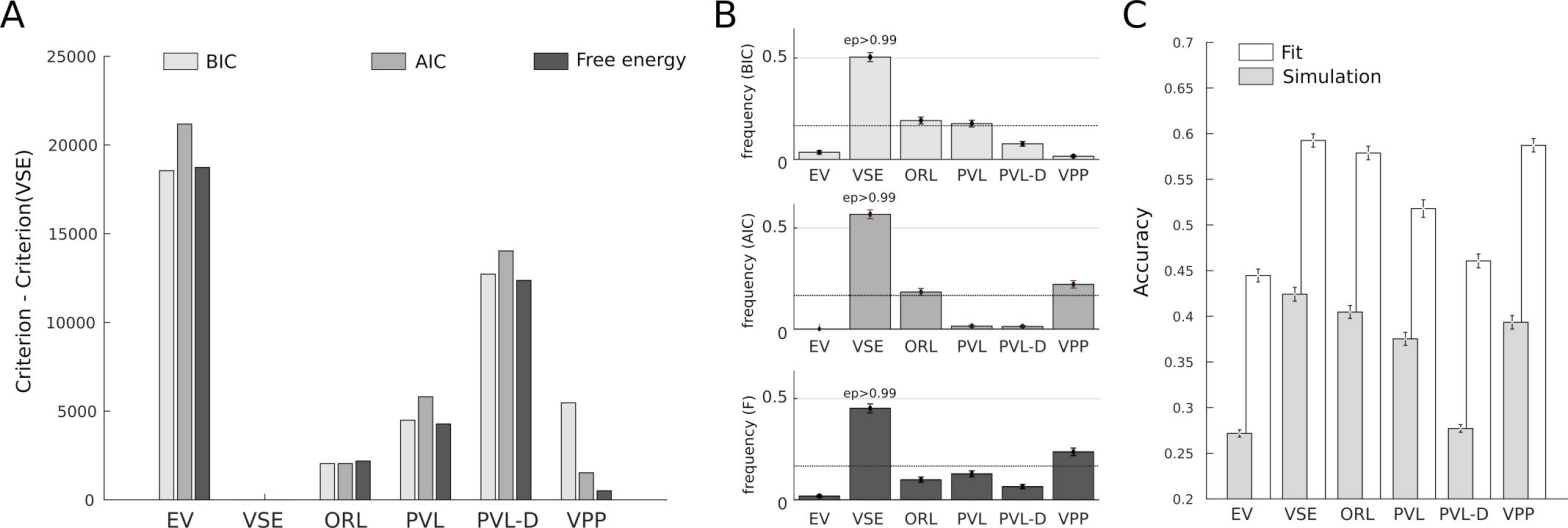
Model comparison. (A) Model comparison treating model attribution as a fixed effect showed that the VSE model outperformed all other models, independently of the penalization for complexity implemented by the different estimators (BIC, AIC or Free Energy). The least difference, observed with the VPP model using Free Energy, still reflected decisive evidence in favor of the VSE model (Bayes Factor > 100)[19]. (B) Bayesian model comparison treating model attribution as a random effect also showed that the VSE model outperformed all other models on the 3 estimators (exceedance probability superior to >0.99 in every case). (C) The VSE model was also the best model for predicting single decision, both for fitted and simulated choice data. For fitted choice data, accuracy refers to choice probabilities as produced by the best-fitting parameters. For simulated choice data, accuracy refers to choice probabilities as produced by the best-fitting parameters based on simulated data (in both cases, accuracy equals 1 if the actual choice corresponds to the highest probability under the model, 0 otherwise).

The comparison of the VSE architecture with the 5 alternative models (described in Methods) was performed using the 504 subjects dataset. First, a fixed-effect analysis comparing summed Bayesian Information Criterion (BIC), Akaike Information Criterion (AIC) and Free Energy (F) metrics over the whole cohort demonstrated decisive evidence in favor of VSE. In order to compare Free Energy (F) with the other metrics, it was transformed to -2*F for this analysis [18]. The difference between the VSE model and other models was everywhere superior to 512 (Fig 2A; the least difference being observed with the VPP model based on the Free Energy estimator). Note that a difference superior to 100 is generally considered as decisive evidence indicating that choosing the second-best fitting model would incur unacceptable information loss [19]. Going further, we performed a Bayesian Group Comparison (Stephan et al., 2009) based on the log-evidence of each model and treating model attribution as a random effect. In order to obtain log-evidences, we transformed AIC and BIC values to -AIC/2 and -BIC/2, respectively (Free Energy natively represents that quantity and takes into account the uncertainty over parameters when penalizing for model complexity). Performed using all available metrics (BIC, AIC, F), this analysis showed that the estimated frequency of the VSE model was in every case superior to 40% and that its approximate exceedance probability (Ep, probability that a given model is the best candidate model to explain the data) was always superior to 0.99 (Fig 2B). Overall, both approaches to model comparison provided overwhelming evidence in favor of the VSE architecture.

Regarding the relationship of model parameters with performance (defined as the number of advantageous minus disadvantageous deck selection), it appeared that value sensitivity was the strongest predictor (ρ = 0.39, p<0.001), followed by *φ* (ρ = -0.24, p<0.001), decay (ρ = 0.23, p<0.001) and temperature (ρ = -0.12, p = 0.006)(S1A Fig). Moreover, although a substantial interindividual variability was observed in SE, the parameter *φ* corresponding to the exploration bonus was significantly superior to 0 (z = 4.78, p<0.001), in line with the existence of directed exploration. A correlation approach then confirmed that this key parameter reflected to which extent participants engaged sequential exploration as assessed by the SE index (Spearman ρ = 0.76, p<0.001). Finally, since the likelihood of observing SE depends on which extent participants exploited the reward structure of the IGT, other parameters also predicted the SE index, but to a much lesser extent (update of exploration weights: ρ = -0.26, p<0.001; value sensitivity: ρ = -0.20, p<0.001; consistency parameter: ρ = 0.13, p = 0.003)(S1B Fig).

### Simulation: Model and parameter recovery

In order to make sure that the advantage of the VSE model reflected a better ability to predict choice data, both qualitatively and quantitatively, we used the 504 sets of parameters associated with each model to simulate 504 *in silico* agents playing the IGT. For each deck, feedbacks were drawn randomly from their corresponding empirical distributions, hence keeping reward contingencies similar across actual and simulated datasets. Then, we applied the exact same fitting procedure to this simulated dataset.

**Fig 3:**
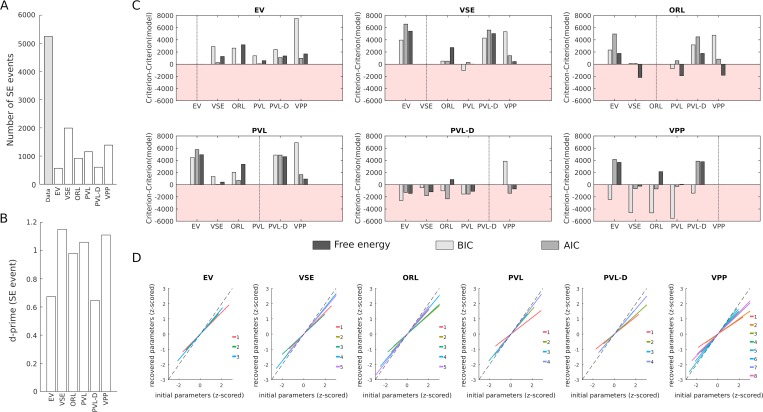
Ability to account for sequential exploration and recovery rates across models. (A) Compared to other models, the VSE model predicted a higher number of sequential exploration (SE) events. (B) The VSE model had also the highest sensitivity to sequential exploration, as shown by a d-prime analysis. (C) The results of the model recovery analysis clearly showed that the EV, VSE and PVL models could be efficiently recovered while the PVL-delta and VPP models showed the worst performance, with the ORL model having an intermediate status. Note that the red areas correspond to situation where a model which was not used to generate the data (indicated by the dashed vertical line) produced better fit than the generator model (see Methods for details). (D) The linear regression curves linking the parameters initially estimated based on participants’ choices and those estimated based on simulated choices showed that the VSE model had the highest parameter recovery performance (i.e minimal deviation from the identity diagonal).

First, we evaluated to which extent each model was able to reproduce participants’ choices using both first-pass and second-pass (i.e simulated) predictions (chance level: 25%, Fig 2C). Again, the VSE model was the most performant model. The first-pass (i.e fit on actual data) reproduced 59.2+/-16% of the choices, whereas the second best model in this respect (VPP) reproduced 58.7+/-17% of the choices. While the difference between VSE and VPP was not significant (z = 0.67, p = 0.50), it must be noted that the VPP has 3 more free parameters which results in a greater chance of overfitting. In this respect, it is interesting to note the difference observed when comparing how the choices derived from simulated data reproduced participants’ choices. Here, the advantage of VSE was clear, with 42.5+/-17% of successful predictions against 39+/-16% for VPP (z = 3.63, p<0.001). Importantly, the VSE model was also the model which predicted the highest number of sequential exploration events (Fig 3A; 1993 against 1389 for VPP, the second model in this respect; raw data: 5247 events) and it was also the most sensitive model according to a d-prime analysis computed over all participants (Fig 3B; 1.15 against 1.11 for the VPP). Note that dependent quadruplets were used for this analysis (ie. 1–4, 2–5, etc.).

Second, we evaluated to which extent each model could be recovered. Low models recovery rates implies that the choice data generated by a given model can be better explained by other models, hence suggesting that there is no specific behavioral signature associated with this model and that the interpretations of the best-fitting parameters should be taken with caution. This computationally intensive analysis (see Methods for details) showed good recovery performance for three models: EV, PVL and VSE (Fig 3C). The advantage for the model actually used to generate the data was decisive in all cases for all estimators, except for the BIC metric, when fitting data generated with the VSE model with the PVL model. This latter exception is very likely due to the fact that the BIC over-penalized model complexity and thus favored the simpler PVL architecture. Moreover, the fact that the highest confusion occurred between the VSE and the PVL models is sensical, given that the “exploitation module” of the VSE model is based on the same accumulation mechanism used by the PVL model. By contrast, the analysis of the three remaining models (ORL, PVL-Delta and VPP) showed that the confusion spanned several models on at least one estimator (e.g. VSE, PVL and VPP performed better to fit ORL-generated data, all models performed better to fit VPP- and PVLDelta-generated data).

Third, we investigated how the parameters estimated from individual choice data could be recovered for each model. Indeed, methodological studies in the field of computational modeling have demonstrated that different combinations of parameters can account for the same sequence of decisions, and that small deviations in parameters values can conversely result in significantly different sequences of decisions, hence impeding the interpretability of best-fitting parameters in some cases. Parameter recovery was assessed by examining how the best-fitting parameters from the second-pass correlated with the best-fitting parameters from the first-pass (i.e that based the actual data). Overall, the recoverability of parameters of VSE was superior to that of other models (mean R = 0.81, range: 0.67–0.95). EV, PVL and ORL also showed good recoverability (EV, mean = 0.76, range: 0.66–0.83; PVL: mean = 0.79, range 0.51–0.94; ORL: mean = 0.77, range = 0.65–0.89), while PVL-delta and VPP were less stable (PVL-delta: 0.71, range: 0.5–0.86; VPP, mean = 0.70, range: 0.41–0.94). In particular, it must be noted that the parameter *φ* reflecting the exploration bonus of the VSE had the highest recoverability (0.95), hence making it a relevant target for the study of inter-individual differences (Fig 3D).

### Aging

In their study (included in the 504 participants dataset analyzed above), Woods and colleagues reported that old and young adults performed equally well on the Iowa Gambling Task but resorted to different strategies. More precisely, old adults appeared to forget more rapidly about outcomes than healthy participants but compensated this forgetting by a better ability to translate what they learned into consistent choice patterns.

**Fig 4:**
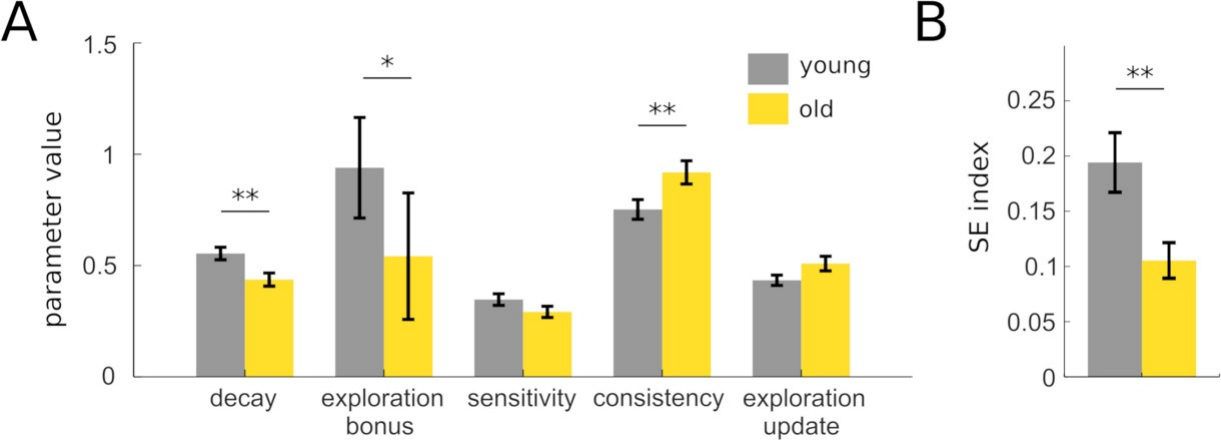
Model-based analysis of IGT behavior in a healthy aging cohort. (A) Using the VSE model as a tool to refine the characterization of age-related changes in risky decision-making, we observed that old and young healthy adults differed on 3 parameters. Relative to young adults, old adults had a lower decay parameter (reflecting a faster forgetting of exploitation weights), a higher consistency (reflecting a more deterministic choice policy) and a lower exploration bonus (reflecting a lower tendency to engage sequential exploration). (B) The age-related reduction in the exploration bonus was reflected into a significantly lower SE index in old as compared to young participants.

Thus, we used this subset of the data to evaluate how well the VSE model could capture heterogeneities in IGT strategies and to validate our modeling approach (Fig 4A). In particular, based on the existing literature, we hypothesized that the exploration bonus should be lower in old as compared to young participants. First, we confirmed that old participants indeed forgot more rapidly than young participants according to the VSE model, as indicated by a lower decay parameter (young: 0.55+/-0.27; old: 0.44+/-0.23; z = 2.75, p = 0.006). Second, the consistency parameter of old participants was indeed higher than that of young participants (young: 0.75+/-0.42; old: 0.92+/-0.41; z = 2.71, p = 0.007). Third and most importantly, young and old participants differed significantly in their *φ* parameter controlling the intensity of directed exploration in the IGT (young:0.94+:-2.14; old: 0.54+/-2.25; z = 2.10, p = 0.036). This latter result paralleled the model-free analysis of SE indexes which also revealed a reduction in directed exploration in the aging group (pattern frequency: young = 16.5+/-14/6%, old = 10.5+/-12.8%; t(151) = 2.61, p = 0.01; Fig 4B) Overall, these results demonstrate the ability of the VSE model to capture age-related changes in directed exploration.

## Discussion

In this study, we uncovered a new choice pattern reflecting the presence of directed exploration within the standard version of the IGT. Indeed, the selection of 4 different decks over 4 consecutive trials—a phenomenon captured by the “sequential exploration index”—largely exceeded chance levels in a group composed of 504 participants, especially in the initial phase of the task. This discovery implies that the IGT can be used to study information-seeking behaviors within risky decision-making contexts. In order to better characterize and quantify this cognitive process within single individuals, we developed a new computational model (VSE, for Value and Sequential Exploration) able to articulate directed exploration with the motivation to optimize gains and losses, using only 5 parameters. The VSE model outperformed the 5 most prevalent models previously used to fractionate the cognitive processes engaged by the IGT, in terms of log-likelihood (penalized for model complexity), prediction accuracy, as well as model and parameter recovery rates. We further demonstrated the potential of this architecture to capture fine-grained differences in IGT behavior between young and old participants. Last but not least, we published the scripts used to generate our results under the form of a user-friendly Matlab toolbox which shall enable the community of researchers and clinicians relying on the IGT as a routine assessment of risky decision-making to report more informative and detailed results with minimal programming and mathematical skills.

In the field of reinforcement-learning, most algorithms are oriented towards normative utility-maximization goals. To do so, they rely heavily upon reward prediction errors, a quantity widely used as a teaching signal enabling step-by-step convergence towards a utility maximum. Yet, likewise all gradient ascent methods, reinforcement-learning algorithms face the risk of reaching only local, rather than global, utility maxima. Directed exploration aims at solving this problem by expanding knowledge about the environment, despite immediate opportunity costs. Thus, directed exploration is particularly valuable when an agent is required to perform numerous decisions within complex or volatile contexts, in which the optimal policy may not be immediately obvious. The IGT is a canonical example of such environment. Indeed, with 4 possible actions leading to highly variable outcomes, the IGT is typically characterized by several successive phases: a “pre-punishment” period during which all decks only produce gains but no losses, a “pre-hunch” period during which punishments start occurring, a “hunch” period during which most healthy participants start feeling that the decks offering the highest average gains actually entails even higher average losses (i.e. deck A and B) and a “conceptual” period during which these participants are able to verbalize that the decks offering small gains (i.e. C and D) are actually the most advantageous ones [20].

Although the term exploration is often applied to choices which are not maximizing utility with respect to a given learning model, the recent rise of predictive coding has completed this conceptualization [21–23]. Indeed, this framework postulates that uncertainty-minimization constitutes a driving principle of our cognitive system alongside utility-maximization, such that modeling the dynamics of exploratory decisions became an important endeavor in the field. Accordingly, recent studies have investigated uncertainty-minimizing strategies in multi-armed bandit tasks using Bayesian methods. In this formalism, options whose mean value is the least precise (or, equivalently, associated with the largest variance) are the best candidates for exploration. The existence of uncertainty-driven exploration was confirmed by some of these studies [9,24,25]. Yet, the type of directed exploration described here does not involve uncertainty computations. Instead, it relies on a simpler recency approach which promotes the exploration of options which have not been selected for a while, independently of the objective uncertainty bound to their pay-offs. This logics can be justified in three ways. First, despite its mathematical elegance, uncertainty-driven exploration does not provide a fully normative solution to the exploration-exploitation trade-off in multi-armed bandits (the process being heavily dependent upon higher-order priors regarding the structure of tasks). Second, the recency method implemented here might still reflect an uncertainty-based mechanism if the subjective uncertainty associated with a given option increases with the duration elapsed since that option was tested for the last time. Third, the seminal study of Daw and colleagues had shown that uncertainty-driven exploration was not useful to describe exploratory patterns in a 4-armed bandit task sharing many commonalities with the IGT [8]. The computational costs associated with uncertainty-tracking may thus become too high within tasks involving more than 3 options. Accordingly, this number recently appeared as an upper limit on the number of stimulus-response mappings whose reliability can be simultaneously monitored by the human executive system [7,26].

The exploration module of our VSE architecture helped going beyond existing models used to account for healthy participants’ decisions in the IGT. Combined with the model-free analysis of the SE index, it expands the heuristic value of the IGT beyond the study of exploitation and reward-seeking behaviors. Moreover, the fact that VSE parameters were on average more recoverable than parameters of previous models will facilitate the interpretation of inter-individual and inter-group differences. Numerous studies which had used the IGT to characterize clinical populations may thus benefit from re-analyzing their data using the toolbox associated with the current paper. Once the trial-by-trial IGT data is converted to the appropriate Matlab format, this toolbox make such re-analysis extremely simple and intuitive, thanks to its compact but informative documentation and its densely commented scripts. With minimal programming knowledge, the six models described hereinabove can be fitted to any standard IGT dataset, compared and evaluated with respect to recoverability and prediction accuracy. These variables as well as other model-free measures (net scores, sequential exploration indices, choice entropy, etc.) can also be calculated, plotted and compared across different groups. While our analyses indicate that the IGT can be suitable to study sequential exploration in conjunction with the VSE model, it must however be emphasized that no modeling approach can circumscribe the non-orthogonal relationship of risk and valence inherent to the structure of the task. In particular, the IGT is not well suited to estimate loss aversion, as implemented by the VPP, PVL and PVL-Delta models. Accordingly, our data showed that the loss aversion parameter had very low recovery rates in the PVL-Delta and VPP models (0.55 and 0.48, respectively) and likely interfered with the value sensitivity parameter in the PVL model (recovery rate: 0.52). Moreover, adding a loss aversion parameter to the VSE model did not improve model fits (see S2 Fig for details). Researchers specifically interested in loss aversion should thus use other paradigms designed to do so [27].

At the neurobiological level, directed exploration likely depends on the prefrontal cortex (PFC), and more particularly on its rostrolateral portion (rlPFC). Indeed, several neuroimaging studies of directed exploration found that the rlPFC is more active during exploratory decisions (Badre et al., 2012; Boorman et al., 2009; Daw et al., 2006). Brain stimulation studies further showed that disrupting or facilitating rlPFC activity can significantly diminish or increase directed exploration, respectively [9,28]. Importantly, disrupting rlPFC activity using continuous theta burst TMS similarly lowered directed exploration and exploration bonuses as assessed by the VSE model in the IGT [29]. This involvement of the rlPFC might also explain the decrease in directed exploration seen in aging individuals, as grey matter density in this area is significantly reduced in old as compared to young adults [30]. Yet, other neural systems certainly interact with the rlPFC to orchestrate information-seeking in reinforcement-learning tasks, including the dmPFC which may control the switch from exploitation to exploration [31]. The prefrontal turn-over of dopamine might also play a pivotal role in regulating directed exploration [32,33], whereas noradrenaline might be involved in the control of random but not directed exploration [34].

In order to further validate our model and illustrate the utility of VSE for the analysis of group differences, we investigated how aging influenced its parameters and more particularly the exploration bonus parameter. The results of this analysis were well aligned with those reported in the study of Wood and colleagues [15], in that the VSE model still evidenced the exacerbated forgetting of previous outcomes in older adults, as well as the reduction in random exploration (i.e increased choice consistency) thought to compensate faster forgetting rates in these participants. More importantly, old adults also displayed a lower exploration bonus than young adults. This effect paralleled the reduction in directed exploration observed when computing directly the frequency of choosing 4 different decks over 4 consecutive trials (SE index). It is also highly consistent with recent papers showing that directed exploration reduces across lifespan [16,17]. Since directed exploration requires the retention of the last few choices made in the task, the phenomenon may be related to the decline of working memory performances sometimes observed in aging cohorts [35].

Beyond the study of cognitive aging, the good recoverability of the VSE model itself and the excellent recoverability of its free parameters constitute two useful features with respect to the development of computational psychiatry. Indeed, the Variational Bayes approach adopted here can be readily combined with the advanced clustering techniques underlying this growing field of research (https://mbb-team.github.io/VBA-toolbox/), which aims at redefining the dimensionality of behavioral impairments across clinical labels in the hope of promoting drug discovery and personalized medicine [36]. To which extent the decomposition of IGT-related behaviors will contribute to this effort remain uncertain, but it has a high potential which could be realized if more clinical teams subscribe to the open-science philosophy by sharing their raw data. Following the initiative of Ahn and colleagues who provided data of stimulant and opiate users [37], addiction research appears as a timely candidate: indeed, large datasets exist for alcohol use [38,39], cannabis use [40], as well as for behavioral addictions such as gambling and eating disorders [41,42].

To conclude, our study leveraged the power of an open “many labs” dataset in order to demonstrate the existence—and characterize the influence—of an overlooked behavior in the IGT. Building on previous work and more particularly on the Prospect Valence Learning (PVL) model [43], the VSE architecture represents not only a quantitative but also a qualitative improvement upon alternative models by shedding light on directed exploration. Besides enabling any experimenter to fit the VSE and its ancestors (EV, PVL, PVL-Delta, VPP, ORL) on IGT data, the toolbox accompanying this paper might be used as an environment to develop even better models in the future. It must be acknowledged that this tool relies heavily on two other open-source packages for Matlab: modeling analyses largely depend on the VBA toolbox by Daunizeau and colleagues [18] whereas visualizations take advantage on the Gramm toolbox by Morel [44]. Last but not least, this study is fully aligned with the ideals of reproducibility and transparency in science: the dataset used is both large and freely available, while the scripts used to generate figures and statistics are available online alongside a clear documentation (https://github.com/romainligneul/igt-toolbox).

## Methods

### Dataset and participants

The dataset comes from a ‘many labs’ initiative grouping 10 studies and containing data from 617 healthy participants [13]. Here, we restricted the analysis to the subset of 7 studies which used the classical 100 trials version of the IGT, resulting in 504 participants (age range: 18–88 years; for the 5 studies with available information about sex: 54% of females). Within this dataset, 153 participants come from a single study on aging [15]. Among these participants, 63 are older adults (61–88 years old; 17 males) and 90 are younger adults (18–35 years old; 22 males) matched in terms of education level and intelligence (WASI vocabulary).

### Sequential exploration index

In order to quantify directed exploration in the IGT, we computed the probability of choosing the 4 different decks during series of 4 consecutive trials. We refer to the frequency of such choice pattern as “SE index”. We used this metrics because the occurrence of such events has a probability of only 9.38% under purely random exploration (note that exploitation makes this probability even smaller by introducing an imbalance in the choice probability of different decks). Although directed exploration is certainty governed by more complex heuristics (resulting in more complex choice patterns), this index was used to ascertain its presence and provide an estimation of its intensity. Inferences about the presence of sequential exploration used independent quadruplets of successive trials (i.e: 1–4, 4–8, etc.), whereas inferences about interindividual differences used dependent quadruplets to maximize sensitivity (i.e: 1–4, 2–5, 3–6, etc.).

### Previous models

Four previous models have been exhaustively and excellently described in a previous publication by Steingroever and colleagues [45]. The ORL model is described in Haines et al. [6]. Therefore, we will only provide a brief overview of their characteristics and then focus mainly on describing the features of the new VSE model.

- The Expected Value (EV) model consists in a simple delta rule allowing for asymmetric consideration of gains and losses when updating the exploitation weight of decks. It has thus a learning rate ɑ (∈ [0,1]) parameter and a reward-punishment asymmetry parameter ⍵ termed “attention weight” (∈ [0,1]).
- The Prospect Valence Learning (PVL) model is a working memory model in which past outcomes are discounted with a decay parameter Δ (∈ [0,1]). Lower decay values imply faster discounting of past outcomes. Outcomes themselves are transformed based on the principles of prospect theory [46]: value sensitivity *ν* and loss aversion ɭ (∈ [0,5]).
- The PVL-delta model applies the same transformation than the PVL model to outcomes, but it uses a delta rule to update exploitation weights and therefore has no decay parameter but a learning rate (as in EV).
- The Value Plus Perseverance (VPP) model is the PVL-delta model extended with a “perserveration module”. This module has 3 parameter: a “persistance after gains” parameter ɛ_gain_ (∈ [0,1]), a “persistance after losses parameter” ɛ_loss_ (∈ [–1,1]), and decay parameter Δ_pers_ (∈ [–1,1]) controlling to which extend past persistance values are discounted. At the decision stage, perseveration values and exploitation weights are combined thanks to an expectancy weight parameter ⍵_Ev_ (∈ [0,1]).
- The ORL model tracks the frequency of positive and negative outcomes using a delta rule using two learning rates (for positive and negative outcome) ranging from 0 to 1. It also tracks separately the magnitude of positive and negative outcomes using the same two learning rate. It is also equipped with a perseveration module characterized by a hyperbolic decay parameter K ∈ [0,242] determining to which extent previously selected decks are attractive, irrespective of their outcome frequency and magnitude. Finally, outcome frequency, magnitude are multiplied by 2 unconstrained parameters and included in a softmax alongside the perseveration values to produce choice probabilities.

All the models described above have in addition a consistency parameter determining to which extend choices are driven by learned values (or any type). This consistency parameter *c* is allowed to fluctuate in the [0,5] interval and is transformed before being used as an inverse temperature parameter β (β = 3^*c*^-1), except for the EV model where c is allowed to fluctuate in the [–2,2] interval and is transformed differently (β = (t/10)^*c*^ with t corresponding to current trial number). In sum, the EV model has 3 parameters, the PVL and PVL-delta models have 4 parameters, the VPP model has 8 parameters and the ORL has 5 parameters. Note that the consistency parameter capture the opposite of “random exploration” (i.e. decision temperature).

### Fitting procedures

A validated toolbox (http://mbb-team.github.io/VBA-toolbox) was used to optimize model parameters [18]. This toolbox relies on a Variational Bayesian (VB) scheme. Compared to non-Bayesian methods, this approach has the advantage of accounting for the uncertainty related to estimated model parameters and of informing the optimization algorithm about prior distributions of parameters’ values. All priors were natively defined as Gaussian distributions of mean 0 and variance 3, which approximates the uniform distribution over the [0–1] interval after a sigmoid transformation. Depending on the range of values in which each parameter was allowed to vary, the sigmoid-transformed parameters were further stretched or shifted to cover different intervals while preserving the flatness of their prior distribution (e.g. “multiplied by 2, minus 1”, to obtain the interval [–1,1]). Model comparison results were replicated using a non-Bayesian model fitting procedure which relied on the standard *fminunc* function of Matlab (line-search algorithm).

All hidden states (i.e values) were initialized at 0 except for exploration weights which were initialized at 1 (since no deck has been sampled at the beginning of the task). The VB algorithm was not allowed to update the initial values for hidden states.

### Model comparison

Comparison of VSE model with the 5 alternatives was first based on a classical fixed-effect analysis comparing summed Bayesian Information Criterion (BIC), Akaike Information Criterion (AIC) and Free energy (F) metrics over the whole group. In this approach, it is classically considered that a difference of 10 units between the models with the lowest and the second lowest criterion value reflects very strong evidence in favor of the model with lowest value (corresponding to a Bayes Factor of 150).

Then, a Bayesian Group Comparison was performed which treated model attribution as a random-effect varying from subject to subject. Also based on BIC, AIC and F, this type of analysis produces an exceedance probability corresponding to the probability that a given model is more likely than any other candidate model (Stephan et al., 2009).

### Model and parameter recovery

There is a growing consensus among computational neuroscientists that evaluating models only based on estimators such as the AIC or BIC is not sufficient [14,47]. The problem is particularly salient when one aims at drawing inferences about cognitive processes from estimated parameters (which is most often the case), because the same choice pattern can sometimes be explained by very different combinations of parameters and because models associated with lower information losses do not always better reproduce qualitative choice patterns. To address these issues and ensure that the VSE model performed equivalently or better than the VPP model in this respect, we performed the simulation and parameter recovery analyses detailed below.

We used the best-fitting parameters of each subject to simulate an artificial decision-maker confronted to the IGT. Simulated choices were generated stochastically according to the consistency parameter, and feedbacks (gains/losses) were drawn from the distributions of feedbacks actually encountered by the participants. Then, we reran model estimations based on these simulated choices, which resulted in a new set of parameters. The quality of parameter recovery for the VSE and VPP models could then be assessed by examining the correlation of this second set of parameters with the parameters initially obtained by fitting real choices. We examined to which extent the initial choices predicted by the model and the choices performed by the simulated participants matched the actual choices of the participants, across models. In this latter analysis, we restricted our statistical inference and compare the VSE model with the second-best fitting model only.

The model recovery analysis consisted in: (i) using each model to simulate 504 series of 100 trials using the parameters distribution obtained after fitting the model on the real dataset; (ii) fitting the 6 candidate models on each of these 6 simulated datasets, hence requiring in 18144 individual fits; (iii) performing a separate model comparison for each of the 6 simulated datasets.

## Supporting information

S1 Fig (A) Relationship between the parameters of the EE model and performance. (B) Relationship between the parameters of the EE model and the SE index.
(TIFF)

S2 Fig Model comparison including a variant of the VSE model using a loss aversion parameter. This model termed VSE+LA, the update of exploitation weights is therefore identical to that of the PVL model, augmented with the sequential exploration module of the VSE. It has therefore 6 parameters. (A) Model comparison treating model attribution as a fixed effect showed that the VSE+LA model did not improve model fit beyond the simpler VSE model which remained the best fitting model, independently of the penalization for complexity (BIC, AIC or Free Energy). However, the VSE+LA model performed better than other alternatives based on the AIC estimator, all but the ORL model based on the BIC estimator and all but the VPP model on the Free Energy estimator. (B) Bayesian model comparison treating model attribution as a random effect confirmed that the VSE+LA model was much less frequent that the simpler VSE model. It is important to note that, in the Iowa Gambling Task, the loss aversion parameter is highly redundant with the value sensitivity parameter: since the magnitude of losses is much higher than that of gains and since the net impact of the nonlinearity introduced by the value sensitivity parameter is proportional to magnitude, a more linear representation of value automatically translates into a stronger avoidance of decks involving high losses. This is why the VSE model only includes a sensitivity parameter.
(TIFF)
