# Supplementary figures and images for: Sequential exploration in the Iowa gambling task: Validation of a new computational model in a large dataset of young and old healthy participants
Source: PLoS Comput Biol. 2019 Jun 13;15(6):e1006989. doi: 10.1371/journal.pcbi.1006989 (PMC6563949; doi:10.1371/journal.pcbi.1006989)

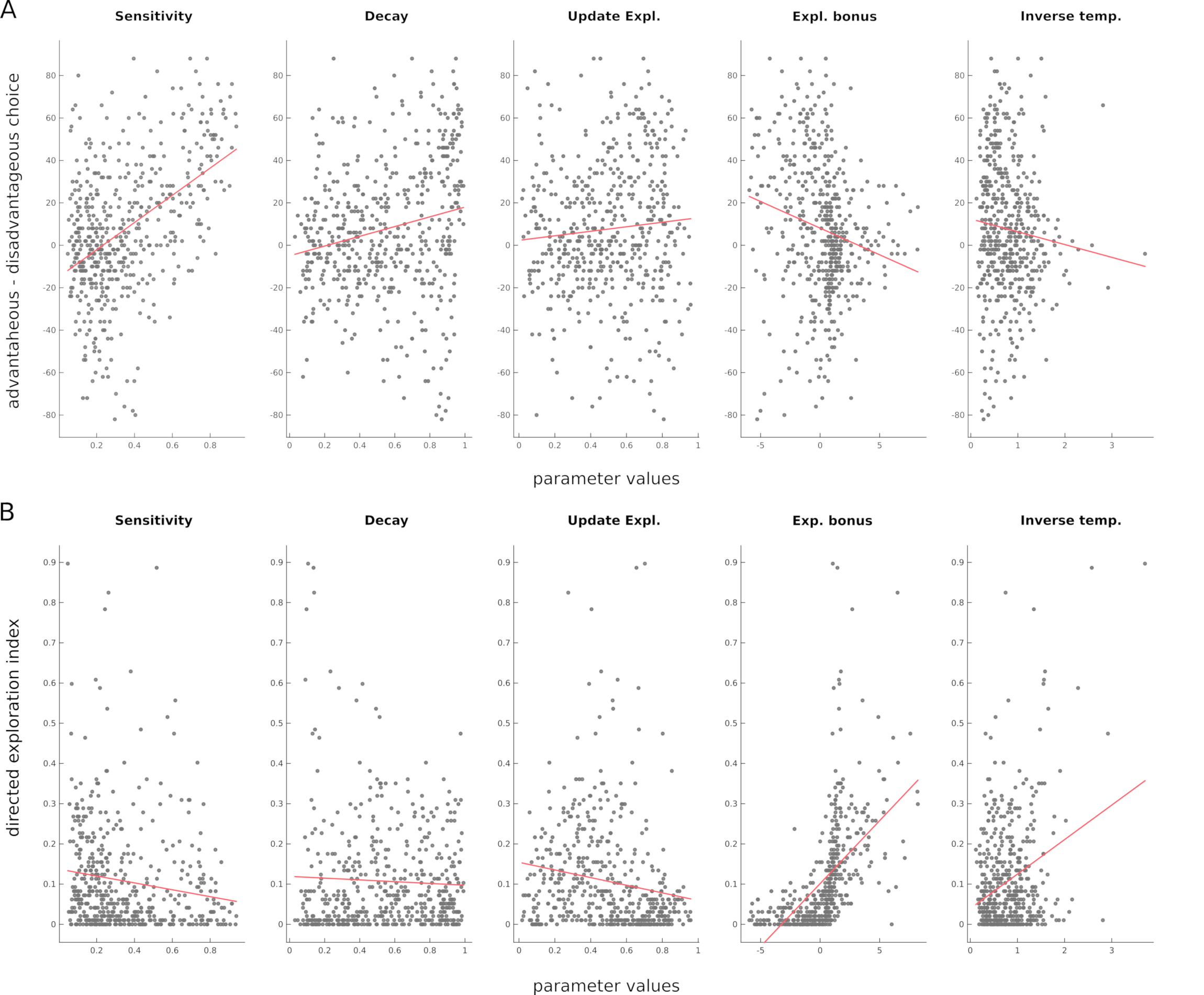

Supplement: S1 Fig — (A) Relationship between the parameters of the EE model and performance. (B) Relationship between the parameters of the EE model and the SE index. (TIFF) [file pcbi.1006989.s001.TIFF]

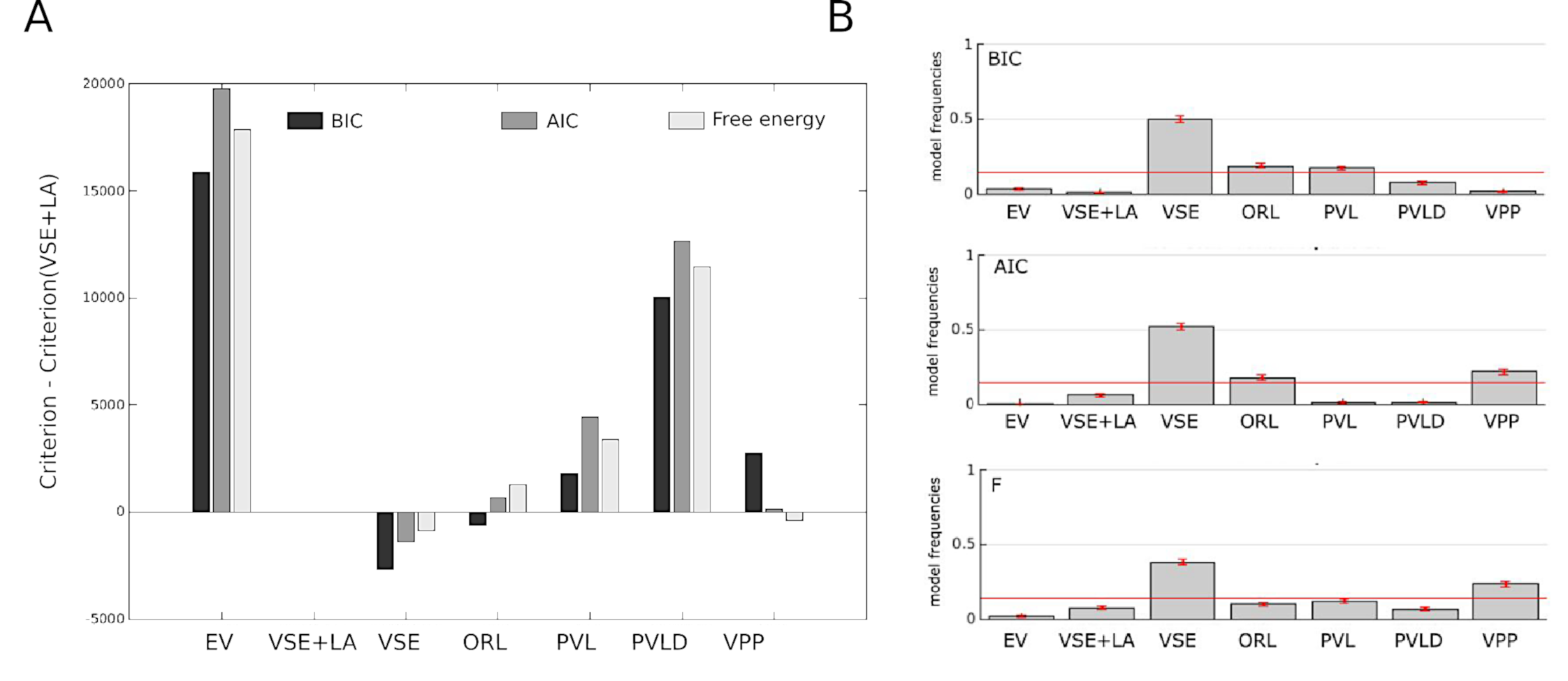

Supplement: S2 Fig — This model termed VSE+LA, the update of exploitation weights is therefore identical to that of the PVL model, augmented with the sequential exploration module of the VSE. It has therefore 6 parameters. (A) Model comparison treating model attribution as a fixed effect showed that the VSE+LA model did not improve model fit beyond the simpler VSE model which remained the best fitting model, independently of the penalization for complexity (BIC, AIC or Free Energy). However, the VSE+LA model performed better than other alternatives based on the AIC estimator, all but the ORL model based on the BIC estimator and all but the VPP model on the Free Energy estimator. (B) Bayesian model comparison treating model attribution as a random effect confirmed that the VSE+LA model was much less frequent that the simpler VSE model. It is important to note that, in the Iowa Gambling Task, the loss aversion parameter is highly redundant with the value sensitivity parameter: since the magnitude of losses is much higher than that of gains and since the net impact of the nonlinearity introduced by the value sensitivity parameter is proportional to magnitude, a more linear representation of value automatically translates into a stronger avoidance of decks involving high losses. This is why the VSE model only includes a sensitivity parameter. (TIFF) [file pcbi.1006989.s002.TIFF]
